# Serum Levels of Tumor Necrosis Factor-α and Loudness Dependence of Auditory Evoked Potentials at Pretreatment and Posttreatment in Patients with Major Depressive Disorder

**DOI:** 10.3390/brainsci9100253

**Published:** 2019-09-26

**Authors:** Bun-Hee Lee, Young-Min Park, Seung-Hwan Lee, Miseon Shim

**Affiliations:** 1 Maum & Maum Psychiatric Clinic, Seoul 02566, Korea; punzza91@naver.com; 2 Department of Psychiatry, Ilsan Paik Hospital, Inje University College of Medicine, Goyang 10380, Korea; lshpss@hanmail.net; 3 Clinical Emotion and Cognition Research Laboratory, Inje University, Goyang 10380, Korea; 4 Department of Medical IT Convergence Engineering, Kumoh National Institute of Technology, Gumi 39177, Korea; miseon@bme.hanyang.ac.kr

**Keywords:** TNF-α, serotonin, source analysis, auditory evoked potentials, LDAEP, sLORETA, major depressive disorder

## Abstract

Background: Proinflammatory cytokines, such as tumor necrosis factor-alpha (TNF-α), are associated with the pathophysiology of major depressive disorder (MDD). Several studies have reported that increased TNF-α might be associated with tryptophan depletion, which eventually could result in MDD. However, other studies revealed that TNF-α increased serotonin firing in raphe. Therefore, whether TNF-α increases or decreases serotonin activity remains unclear. Here, we aimed to determine the relationship between serum TNF-α level and central serotonergic activity using the loudness dependence of auditory evoked potentials (LDAEP) and standardized low-resolution brain electromagnetic tomography (sLORETA), as well as to evaluate the effects of antidepressants on TNF-α levels. Methods: LDAEP, serum TNF-α level, and depression severity were measured in 64 MDD outpatients pre and post 3 months of treatment. Results: Pretreatment TNF-α levels were negatively correlated with the pretreatment N1 sLORETA-LDAEP, P2 sLORETA-LDAEP, and N1/P2 sLORETA-LDAEP (*p* < 0.05). In multiple regression analysis for N1/P2 sLORETA-LDAEP, lower N1/P2 sLORETA-LDAEP was significantly related to higher TNF-α (CE = −0.047, *p* = 0.017) when all subjects were dichotomized based on the median TNF-α level (7.16 pg/mL) into pretreatment low- and high-TNF-α groups. In addition, the pretreatment Beck Depression Inventory, P2 LDAEP, and N1/P2 sLORETA-LDAEP were greater in the high-TNF-α groups than in the low-TNF-α groups (*p* < 0.05). Moreover, the posttreatment TNF-α level was significantly decreased compared to the pretreatment TNF-α level (*z* = −2.581, *p* = 0.01). However, the posttreatment TNF-α levels were not associated with posttreatment LDAEP. Conclusions: Higher TNF-α level is associated with decreased LDAEP, which could indicate compensatory elevation of central serotonin activity in outpatients with MDD, although this effect disappeared and TNF-α level was reduced after three months of antidepressant treatment.

## 1. Introduction

The monoamine hypothesis is considered as the main pathophysiology underlying a major depressive disorder (MDD). However, various hypotheses have emerged recently, of which one states that proinflammatory cytokines such as tumor necrosis factor-alpha (TNF-α) play a significant role in MDD. Recent meta-analysis revealed higher levels of TNF-α in patients with MDD than those in healthy subjects [1]. Another hypothesis is related to the glutamate system [2]. Several studies have found network dysfunction and altered brain levels of glutamate in depression, and the findings of these studies suggest that ketamine treatment may be both effective and rapid in patients with treatment-resistant MDD [3,4]. These findings are in contrast to the monoamine hypothesis and have led to the recent emergence of an integrative hypothesis that includes monoamine, glutamate, and the cytokine theory of depression [5].

Stress activates the hypothalamic–pituitary–adrenal (HPA)-axis and leads to secretion of cortisol, which eventually is responsible for secretion of proinflammatory cytokines such as TNF-α from the microglial cells. In addition, TNF-α can be produced from a variety of cells such as fibroblasts, endothelial cells, neurons, mast cells, and adipose tissue [6]. The inflammatory process can also activate the production of peripheral TNF-α [6]. TNF-α reaches the brain through some immune-mediated pathways and transfers the signals from the periphery to the brain [7]. The elevation of cytokines and cortisol in the central nervous system (CNS) enhances tryptophan metabolism, which is assimilated into kynurenine under the persisting stress, rather than being metabolized into serotonin [5]. Kynurenine is eventually metabolized into quinolinic acid, which is a glutamate agonist and functions as a neurotoxin in the brain [5].

Nevertheless, in contrast, few previous studies suggested that TNF-α elevation increases the serotonin activity or levels [6,7]. That is, it remains unclear whether TNF-α increases or decreases the serotonin activity in patients with depression. Hence, the present study aimed to determine the relationship between the TNF-α level and central serotonergic activity as well as to evaluate the effects of antidepressants on TNF-α.

The loudness dependence of auditory evoked potentials (LDAEP), which is calculated using the amplitude of event-related potentials such as N100 and P200 induced by auditory stimuli, has recently been used to measure the central serotonergic activity [8]. Some clinical and animal studies have reported that the LDAEP is a reliable marker of the central serotonergic activity in psychiatric disease such as MDD, although other studies in humans have revealed inconsistent findings [9,10,11]. Few investigators have found that LDAEP is inversely correlated with the central serotonergic activity [12]. The present study used the LDAEP to identify the relationship between TNF-α and central serotonergic activity, as well as to evaluate the effects of antidepressants on TNF-α.

## 2. Materials and Methods

### 2.1. Subjects and Study Design

In total, 64 outpatients aged between 18 and 65 years, who met the DSM-IV Text Revision criteria for MDD, have been recruited at Ilsan Paik Hospital, Korea. The inclusion criteria for participation of these patients were: (1) Having total Beck Depression Inventory (BDI) and Hamilton Depression Rating Scale (HAMD) scores exceeding 19 or 17, respectively, prior to the treatment, and (2) no history of antidepressant administration during the 12 weeks before their first visit to our hospital. The exclusion criteria were: The presence of a (1) previous hypomanic episode, (2) high suicide risk, (3) history of brain trauma or organic brain disease, or (4) neurological disease. Depression severity was assessed using the HAMD and BDI scores at baseline and at 3 months. The serum TNF-α level and LDAEP were also measured at baseline. In addition, the follow-up serum TNF-α and LDAEP were remeasured in 22 subjects after the 3-month antidepressant monotherapy (escitalopram, paroxetine, and sertraline), as the remaining patients did not wish to undergo follow-up blood sampling. These data were included in the Korean MDD cohort for suicide prevention (KOMDD study) [13].

The study protocol was approved by the Ethics Committee of Ilsan Paik Hospital, and written informed consent to participate was obtained from all patients before commencing the investigations.

### 2.2. Measurements of Serum TNF-α Level

Blood samples for measuring serum TNF-α level were collected between 8:00 a.m. and 9:00 a.m., following an overnight fast by venipuncture, and were added into the anticoagulant-free vacuum tubes. The samples were allowed to clot for 30 min at room temperature, and were then centrifuged at 2000 rpm for 15 min. Serum was separated and aliquots were stored at −70 °C until further analysis. The serum TNF-α level was evaluated at baseline and after 3 months of treatment.

Serum TNF-α level was analyzed using enzyme-linked immunosorbent assay (ELISA) kits (Human Magnetic Luminex Screening Assay, R&D Systems, Minneapolis, MN, USA). Each assay was performed in duplicate. The actual concentration of each sample was calculated using the five-parameter-fit logistic-curve equation. The ELISA plate readings were recorded using a microplate reader (VersaMax, Molecular Devices, Sunnyvale, CA, USA).

### 2.3. Electroencephalography Methods

All subjects were seated in a comfortable chair in a sound-attenuated room. They were asked to keep their eyes open throughout the testing, fixated on a pointer displayed on the monitor. The auditory processing comprised 1000 stimuli with an interstimulus interval of 500–900 ms. Tones at 1000 Hz and with a duration of 80 ms (with 10-ms rise and fall times) were generated by E-Prime software (Psychology Software Tools, Pittsburgh, PA, USA), and presented in a randomized order at 5 intensities (55, 65, 75, 85, and 95 dB SPL) via headphones (MDR-D777, Sony, Tokyo, Japan). Electroencephalography data were recorded from 64 scalp sites using silver/silver chloride electrodes according to the International 10–20 system (impedance <10 K) using an Auditory Neuroscan NuAmp amplifier (Compumedics USA, El Paso, TX, USA). Data were collected at a sampling rate of 1000 Hz after passing the signals through a bandpass filter from 0.5 to 100 Hz. In addition, four electrodes were used to measure the horizontal and vertical electrooculograms.

Data were reanalyzed using Scan 4.3 software with a bandpass filter from 1 to 30 Hz, and the ocular contamination was removed using standard blink correction algorithms [14]. Event-related potential sweeps comprising artifacts exceeding 70 mV were rejected at all electrode sites. For each intensity and subject, the N1 (negative-most amplitude between 80 and 130 ms after the stimulus) and P2 (positive-most peak between 130 and 230 ms after the stimulus) peaks were then determined at the Cz electrode. The peak-to-peak N1/P2 amplitudes were calculated for the five stimulus intensities, and the LDAEP was calculated as the slope of the linear regression curve.

### 2.4. Source LDAEP Analysis

On the basis of the averaged scalp-recorded electric potential, standardized low-resolution brain electromagnetic tomography (sLORETA) was used to estimate the current densities [15]. The sLORETA technique evaluates the standardized source current density by using the realistic three-shell head model based on the Montreal Neurological Institute (MNI) 152 template provided by the Brain Imaging Center of the MNI, under the assumption that the activity at any single neuron will be strongly synchronized to those of its closest neighbors [16]. The solution space is restricted to the cortical gray matter and hippocampus of the head model and partitioned into 6239 voxels at a spatial resolution of 5 mm. Anatomical labels, such as Brodmann areas (BAs), are based on an appropriate transformation from the MNI space to the Talairach space [17]. The loudness dependence of the source activity (source LDAEP) was determined by calculating the current source densities for each subject and each sound pressure level. Two electrodes (M1 and M2) were not used in the sLORETA analysis as their locations are not supported by the sLORETA software. The calculated standardized current density was averaged between 60 and 240 ms post-stimulus from the primary auditory cortex (BA41), in accordance with previous studies [18,19]. We calculated the values of current density for the left, right, and averaged data from both hemispheres over the voxels beneath the primary auditory cortex. The source LDAEP was calculated as the slope of the linear regression of current density of BA41 for the five stimulus intensities.

### 2.5. Statistical Analysis

The Kolmogorov–Smirnov test was used to check whether the clinical variables were normally distributed. Correlation analysis using Pearson’s and Spearman’s correlations was carried to identify the significant correlations between clinical variables and the LDAEP and TNF-α level. All subjects were dichotomized according to the median TNF-α level (7.16 pg/mL) into baseline low- and high-TNF-α groups. Student’s t-test, Mann–Whitney U-test, and the Chi-squared test were used to compare the clinical variables between the two groups. For multiple regression analysis of the relationship between TNF-α and LDAEP, all subjects were dichotomized according to the median TNF-α level (7.16 pg/mL) into baseline low- and high-TNF-α groups. The pre- and posttreatment clinical variables, LDAEP and TNF-α, in the MDD patients were compared by paired t-tests or Wilcoxon’s signed ranks test. All tests were two-tailed, and the cutoff for significant group differences was *p* < 0.05. The statistical analysis was performed using the SALT 2.5 software packages(Eugene, Seoul, Republic of Korea).

## 3. Results

In total, 64 subjects (14 males and 50 females) aged 40.47 ± 13.71 years (mean ± SD) were evaluated in this study. The age, sex distribution, and baseline scores on the BDI, HAMD, LDAEP values, and TNF-α levels are presented in Table 1.

When comparing the clinical variables and LDAEP between pretreatment low- and high-TNF-α groups according to the median TNF-α level (7.16 pg/mL, Table 2), the BDI scores in the high-TNF-α group were significantly greater than those in the low-TNF-α group (*p* < 0.05). In addition, the average P2 LDAEP, left N1 sLORETA-LDAEP, left P2 sLORETA-LDAEP, average P2 sLORETA-LDAEP, left N1/P2 sLORETA-LDAEP, and average N1/P2 sLORETA-LDAEP in the high- TNF-α groups were significantly lower than those in the low-TNF-α groups (*p* < 0.05). In multiple regression analysis for pretreatment N1/P2 sLORETA-LDAEP, lower pretreatment N1/P2 sLORETA-LDAEP was significantly related with pretreatment of high-TNF-α groups (CE(Coefficient) = −0.047, *p* = 0.017, Table 3).

Pretreatment TNF-α was negatively correlated with the pretreatment average N1 sLORETA-LDAEP (*r_s_*= −0.248, *p* = 0.048), left P2 sLORETA-LDAEP (*r_s_*= −0.25, *p* = 0.046), left N1/P2 sLORETA-LDAEP (*r_s_*= −0.257, *p* = 0.040), and average N1/P2 sLORETA-LDAEP (*r_s_*= −0.258, *p* = 0.040).

**Figure 1:**
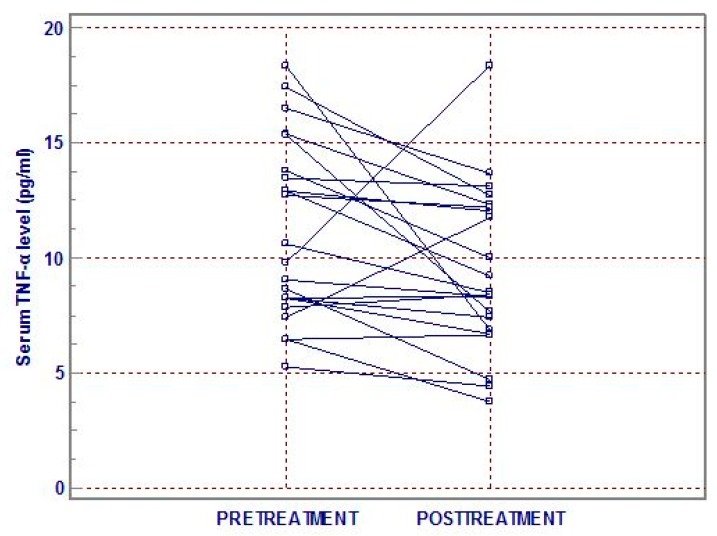
Tumor necrosis factor-alpha (TNF-α) level at pretreatment and posttreatment.

Pretreatment and posttreatment TNF-α levels (*n* = 22) were compared with each other. Each mean TNF-α level was, respectively, 11.14 ± 3.89 and 9.41 ± 3.54 pg/mL, and significantly differed from each other (*z* = −2.581, *p* = 0.01; Figure 1). However, the pretreatment TNF-α levels were not associated with the treatment response as per HAMD and BDI.

## 4. Discussion

The present study revealed three main findings. First, posttreatment TNF-α was significantly decreased compared to the pretreatment TNF-α level. Second, pretreatment TNF-α levels were negatively correlated with the pretreatment N1/P2 sLORETA-LDAEP, although the former was not correlated with pretreatment LDAEP. In multiple regression analysis for pretreatment N1/P2 sLORETA-LDAEP, lower pretreatment N1/P2 sLORETA-LDAEP was also significantly related with the pretreatment high-TNF-α group. Third, pretreatment P2 LDAEP level in the pretreatment high- TNF-α group was significantly lower than that in the pretreatment low-TNF-α group.

TNF-α levels were significantly reduced compared to baseline levels after antidepressant treatment in patients with MDD in the present study. Some evidence revealed that several cytokines are elevated in patients with MDD [20], with proinflammatory cytokines including TNF-α which plays a major role in MDD [7]. Few studies have reported the TNF-α levels to be associated with the severity of MDD and the degree of treatment resistance [1,21]. Other studies found that anti-TNF-α treatment can directly alleviate the depressive state [22,23]. Consistent results have also been found in animal studies, with the TNF-α knockout mouse presenting an antidepressant-like phenotype [20]. The present results are therefore in accordance with the findings of these previous studies.

Another finding of the present study was that in multiple regression analysis for pretreatment N1/P2 sLORETA-LDAEP, lower pretreatment N1/P2 sLORETA-LDAEP was significantly related with the pretreatment high-TNF-α group. According to the cytokine hypothesis, proinflammatory cytokines, such as TNF-α, stimulate indoleamine dioxygenase (IDO) under an activated HPA axis [24]. This IDO results in tryptophan converting into kynurenine rather than serotonin, which further reduces the level of serotonin [25]. Moreover, kynurenine is transformed into a glutamate agonist, that of quinolinic acid, which exerts cerebrotoxic and depressogenic effects [24]. Eventually, the decreased serotonin and increased quinolinic acid levels induce depression. Thus, this hypothesis was not in accordance with the present findings of the central serotonin activity being higher in the high-TNF-α group. However, some investigators found that TNF-α enhances serotonin transporter (SERT) activity by increasing the SERT mRNA levels [26]. In addition, the proinflammatory cytokines also elevate raphe firing and extracellular serotonin [6]. Thus, the hypothesis that proinflammatory cytokines such as TNF-α reduce the serotonin levels seems to be too simplistic.

Few investigators have hypothesized that TNF-α is able to increase the serotonin levels in the synaptic cleft as long as the uptake activity via SERT is intact [6]. They also proposed that the risk of MDD can increase when these homeostatic mechanisms are overactivated [6]. Thus, higher serotonergic activity in the high-TNF-α group, in the present study, could be attributed to an excessive compensatory response with relatively intact homeostasis in our MDD subjects with mild–moderate severity compared to that in patients with severe MDD or admitted MDD. Alternatively, our subjects were all outpatients and had a mean HAMD score of 17.9, which suggests their depression severity was mild-to-moderate. Thus, it is hypothesized that the compensatory response might be increased, thus resulting in higher serotonergic activity in patients with mild-to-moderate MDD or relatively preserved function, whereas this response eventually may get exhausted with reducing the serotonergic activity in the severe MDD of admitted patients. In addition, the low-TNF-α group seemed to be different from the high-TNF-α group, which indicates that MDD comprises heterogeneous subgroups [10].

Several studies have investigated the effects of antidepressants on TNF-α; however, the results still revealed contradictory findings like decreased, increased, or unchanged TNF-α levels after antidepressant treatment [27,28,29]. In the present study, serum TNF-α significantly decreased after the antidepressant treatments. Interestingly, the posttreatment TNF-α levels were not associated with posttreatment LDAEP, unlike the pretreatment relationship between them in the present study. Presumably, the state of increased pretreatment TNF-α and compensatory serotonergic activity was resolved, and the original equilibrium state was retained due to the antidepressant treatment.

The LDAEP has been widely studied in the field of psychopathology, for its relationships with suicide and treatment responses, and as a biological marker associated with serotonin, since Hegerl and Juckel first discovered this noninvasive method [30]. However, while animal studies have consistently demonstrated an inverse relationship between LDAEP and central serotonergic activity, the studies involving humans have produced less consistent findings [9]. Several studies have reported that the LDAEP was more useful in predicting the antidepressant treatment response and as a central serotonergic marker. In addition, our previous study found that the LDAEP value did not differ between the MDD patients and healthy controls, indicating that MDD patients represent a heterogeneous group, and reflecting that the monoamine hypothesis is not fully applicable in clinical populations [10]. The present study has revealed that central serotonergic activity differs according to the value of TNF-α. This suggests that MDD has varying degrees of serotonergic activity being associated with homeostasis or resilience in each individual. Some investigators have assumed that in the setting of sustained overactivity of the regulatory circuit, an exaggerated or hypersensitive compensatory response may result in an agitated, mood-reactive, ruminative depressive state in some patients, whereas failure to initiate or maintain an adequate compensatory response may lead to anergy, psychomotor retardation, apathy, and mood nonreactivity in other patients [31].

This study was subject to several limitations. The sample was relatively small and it did not include healthy controls. In addition, other cytokines were not analyzed in this study. Nevertheless, this study supports that pretreatment TNF-α levels are associated with central serotonergic activity, and antidepressant treatment can reduce the pretreatment TNF-α levels in patients with MDD. Moreover, despite TNF-α being produced in a circadian rhythm-manner and different sleep patterns being observed among the patients, these factors were not considered while designing the experiments. However, blood samples for measuring serum TNF-α level were collected between 8:00 a.m. and 9:00 a.m., following an overnight fast to reduce a bias.

## Figures and Tables

**Table 1:** Demographic and clinical variables in 64 major depressive disorder (MDD) patients at baseline.

Variable	Subjects (*n* = 64)
Age (years) Sex (male/female) ^a^ Total BDI score Total HAMD score N1 LDAEP P2 LDAEP N1/P2 LDAEP N1 LORETA-LDAEP (Lt) N1 LORETA-LDAEP (Rt) N1 LORETA-LDAEP (Av) P2 LORETA-LDAEP (Lt) P2 LORETA-LDAEP (Rt) P2 LORETA-LDAEP (Av) N1/P2 LORETA-LDAEP (Lt) N1/P2 LORETA-LDAEP (Rt) N1/P2 LORETA-LDAEP (Av) TNF-α (pg/mL)	40.47 ± 13.71 14/50 28.39 ± 10.77 17.94 ± 5.05 –0.52 ± 0.67 0.77 ± 0.76 1.28 ± 0.87 0.069 ± 0.14 0.092 ± 0.15 0.081 ± 0.12 0.026 ± 0.093 0.038 ± 0.12 0.032 ± 0.091 0.039 ± 0.084 0.052 ± 0.095 0.046 ± 0.074 8.81 ± 5.92

^a^ Chi-square test. BDI = Beck Depressive Inventory; HAMD = Hamilton Depression Rating Scale; N1 = indicating that it is the first peak and is negative; P2 = indicating that it is a positive peak which follows N1; LDAEP = loudness dependence of auditory evoked potentials; sLORETA = standardized low-resolution brain electromagnetic tomography; tumor necrosis factor-alpha (TNF-α); Av = average; Lt = left; and Rt = right. Data are mean ± SD values.

**Table 2:** Comparison of demographic and clinical variables between low- and high-TNF-α groups.

Variable	Low-TNF-α Group (*n* = 32)	High-TNF-α Group (*n* = 32)	*p*
Age (years)	38.66 ± 11.91	42.28 ± 15.28	0.29
Sex (male/female) ^a^	6/26	8/24	0.55
Total BDI score	25.09 ± 11.62	31.69 ± 8.85	0.013 ^*^
Total HAMD score	17.41 ± 4.38	18.47 ± 5.66	0.40
N1 LDAEP	1.12 ± 0.84	0.93 ± 0.78	0.36
P2 LDAEP	0.96 ± 0.81	0.57 ± 0.68	0.042 ^*^
N1/P2 LDAEP	1.44 ± 0.89	1.13 ± 0.83	0.15
N1 LORETA-LDAEP (Lt)	0.099 ± 0.14	0.039 ± 0.13	0.034 ^*^
N1 LORETA-LDAEP (Rt)	0.10 ± 0.13	0.082 ± 0.16	0.22
N1 LORETA-LDAEP (Av)	0.10 ± 0.12	0.061 ± 0.12	0.062
P2 LORETA-LDAEP (Lt)	0.059 ± 0.097	0.0063 ± 0.078	0.012 ^*^
P2 LORETA-LDAEP (Rt)	0.063 ± 0.13	0.013 ± 0.10	0.10
P2 LORETA-LDAEP (Av)	0.061 ± 0.10	0.0037 ± 0.070	0.012 ^*^
N1/P2 LORETA-LDAEP (Lt)	0.069 ± 0.086	0.0084 ± 0.070	0.008 ^**^
N1/P2 LORETA-LDAEP (Rt)	0.069 ± 0.090	0.035 ± 0.098	0.066
N1/P2 LORETA-LDAEP (Av)	0.069 ± 0.076	0.022 ± 0.065	0.005^**^

Comparisons were made by independent t test or Mann–Whitney U-test. ^a^ Chi-square test. BDI = Beck Depressive Inventory; LDAEP = loudness dependence of auditory evoked potentials; sLORETA = standardized low-resolution brain electromagnetic tomography; Av = average; Lt = left; and Rt = right. Data are mean ± SD values; * *p* < 0.05; and ** *p* < 0.01.

**Table 3:** Multiple regression analysis for pretreatment average N1/P2 sLORETA-LDAEP.

Variables	CE	SE	t	*p*-Value
Intercept	0.019	0.04	0.47	0.64
Age	0.00079	0.00067	1.18	0.24
Sex	0.03	0.022	1.39	0.17
BDI	–0.0002	0.0009	–0.23	0.82
TNF-α ^a^	–0.047	0.019	–2.45	0.017^*^

^a^ Categorical variable (All subjects were dichotomized according to the median TNF-α level into pretreatment low- and high-TNF-α groups); *p < 0.05; CE = coefficient; SE = standard error; BDI = Beck Depression Inventory.
